# Supplementary figures and images for: Long-Term Memory Formation in Drosophila Depends on the 3′UTR of CPEB Gene orb2
Source: Cells. 2023 Jan 14;12(2):318. doi: 10.3390/cells12020318 (PMC9856895; doi:10.3390/cells12020318)

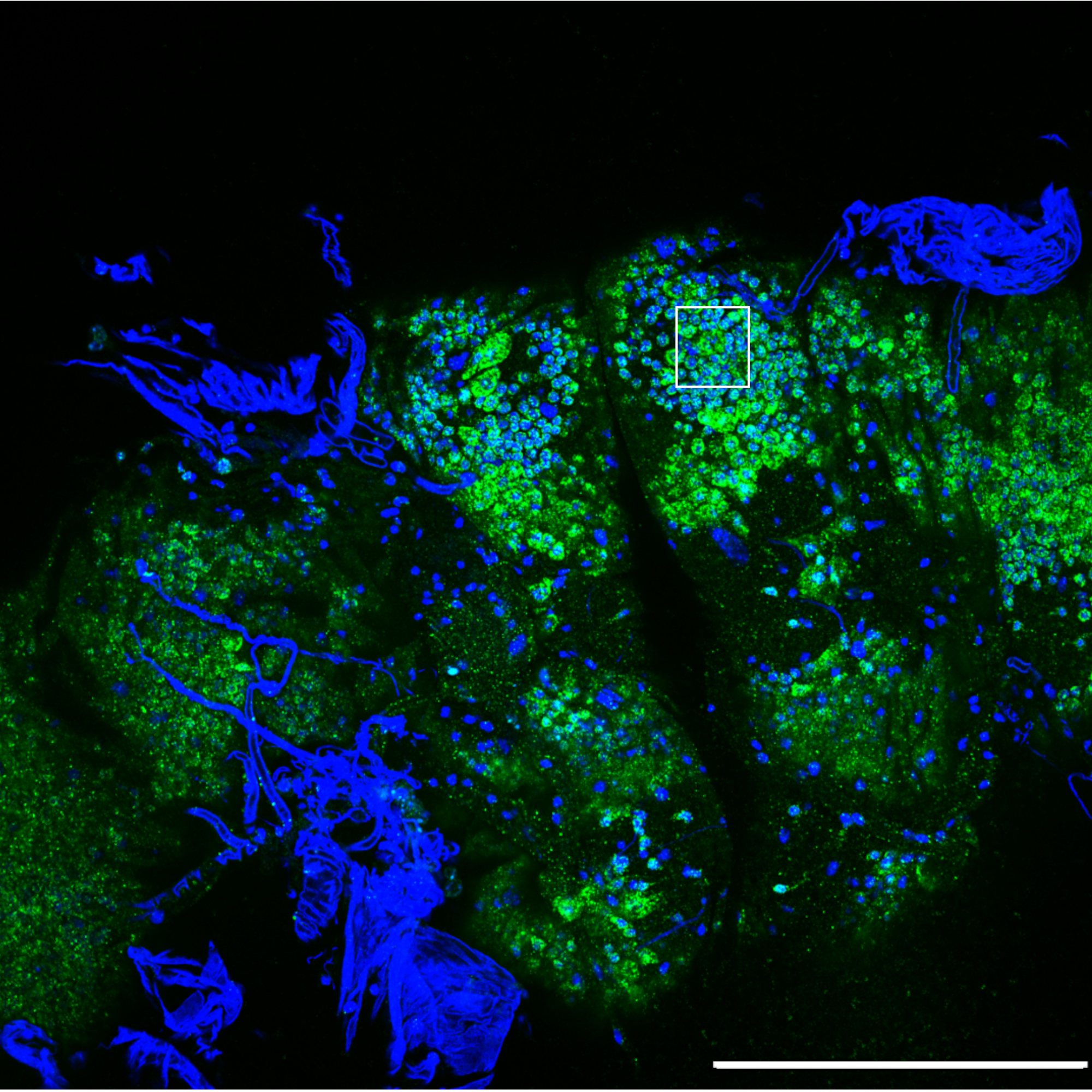

Supplement: Supplementary file 1 [file cells-12-00318-s001.zip › Figure S1.tif]

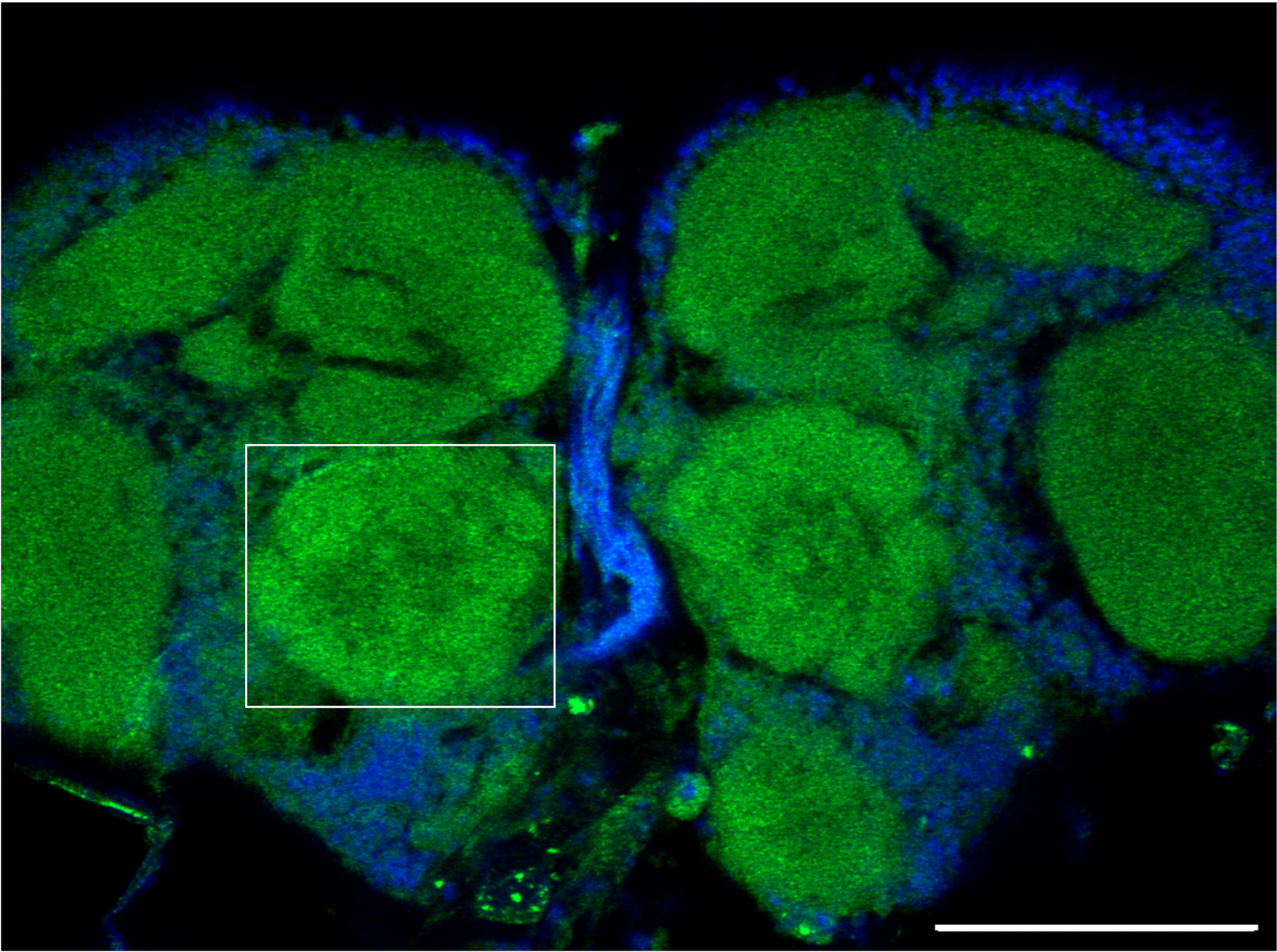

Supplement: Supplementary file 1 [file cells-12-00318-s001.zip › Figure S2.tif]

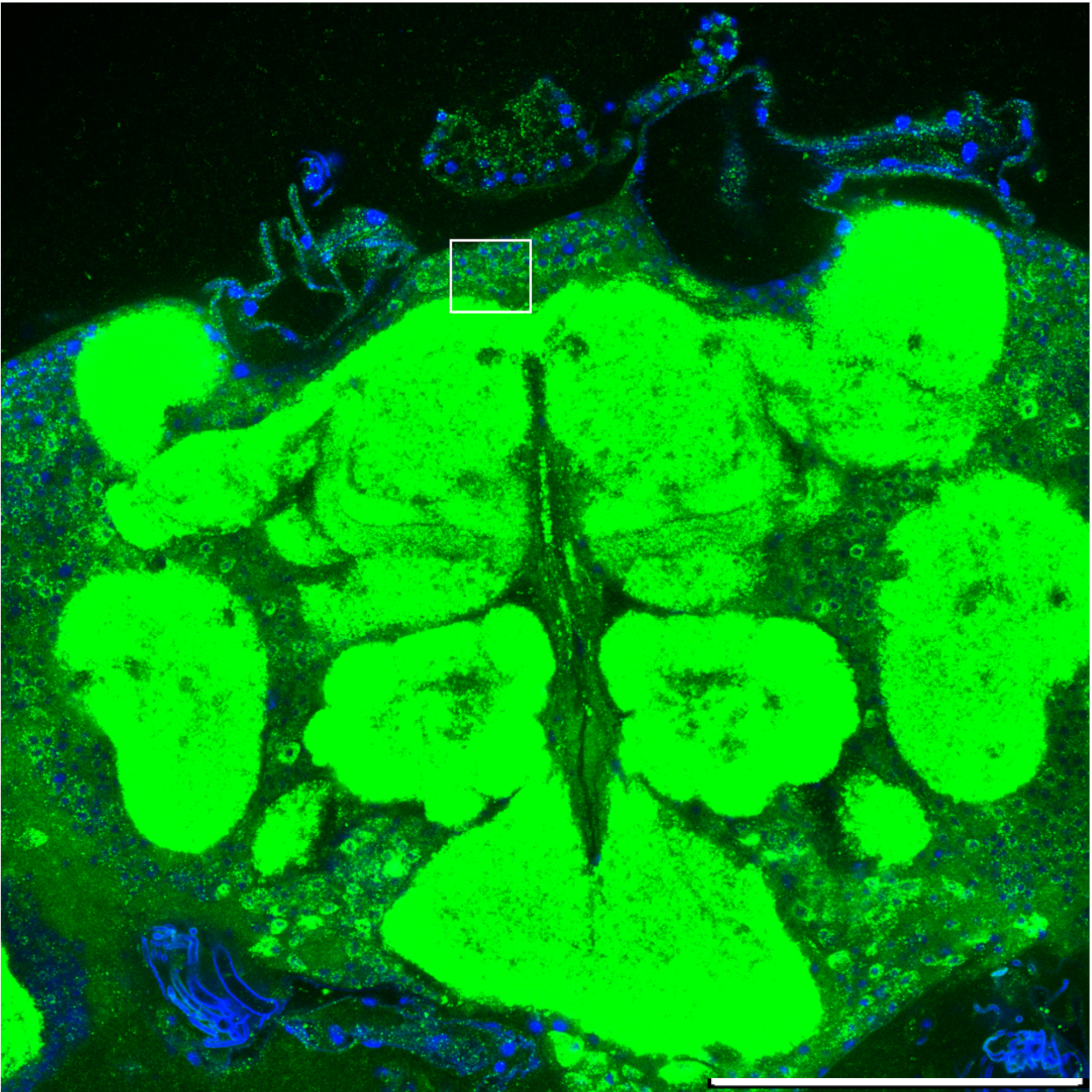

Supplement: Supplementary file 1 [file cells-12-00318-s001.zip › Figure S3.tif]

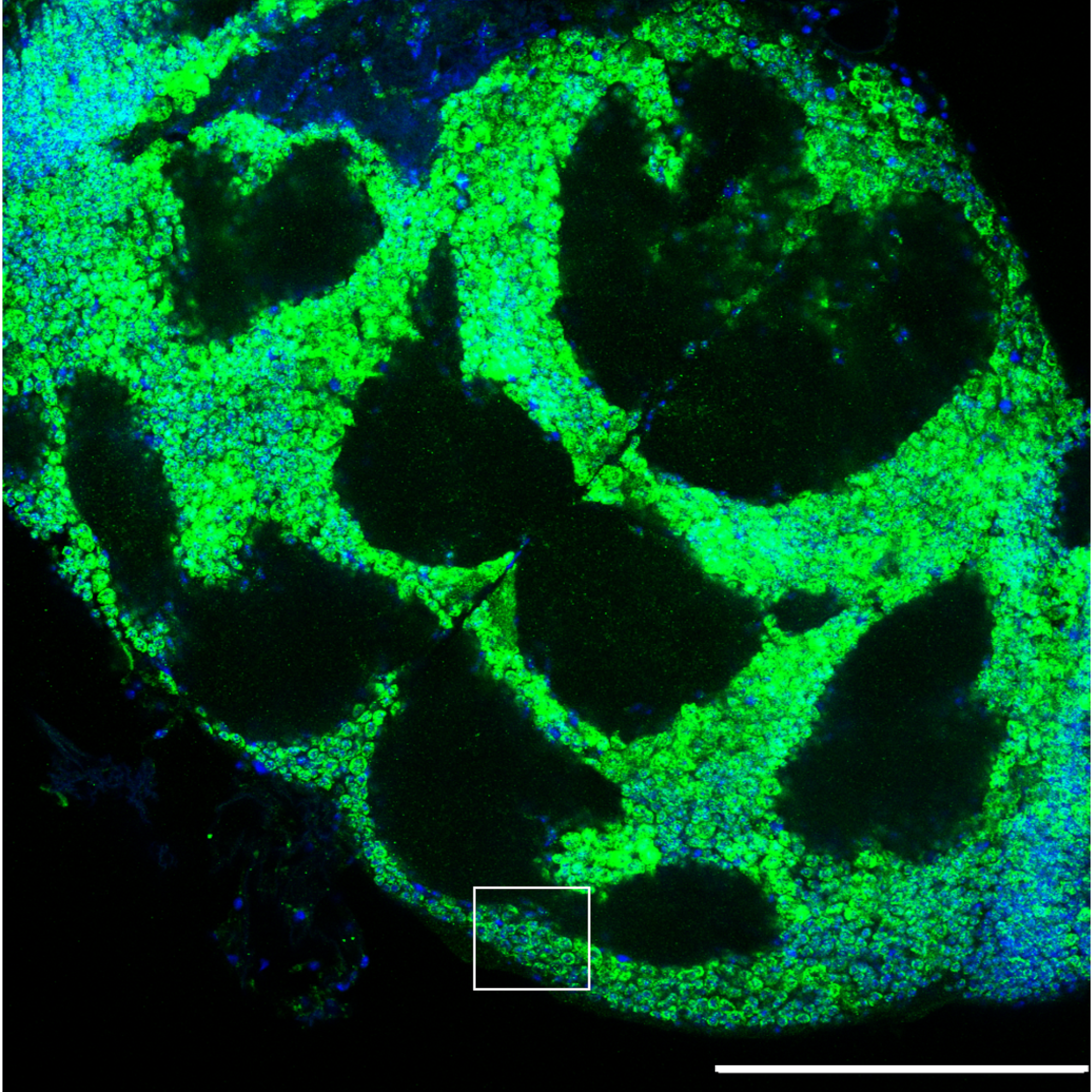

Supplement: Supplementary file 1 [file cells-12-00318-s001.zip › Figure S4.tif]

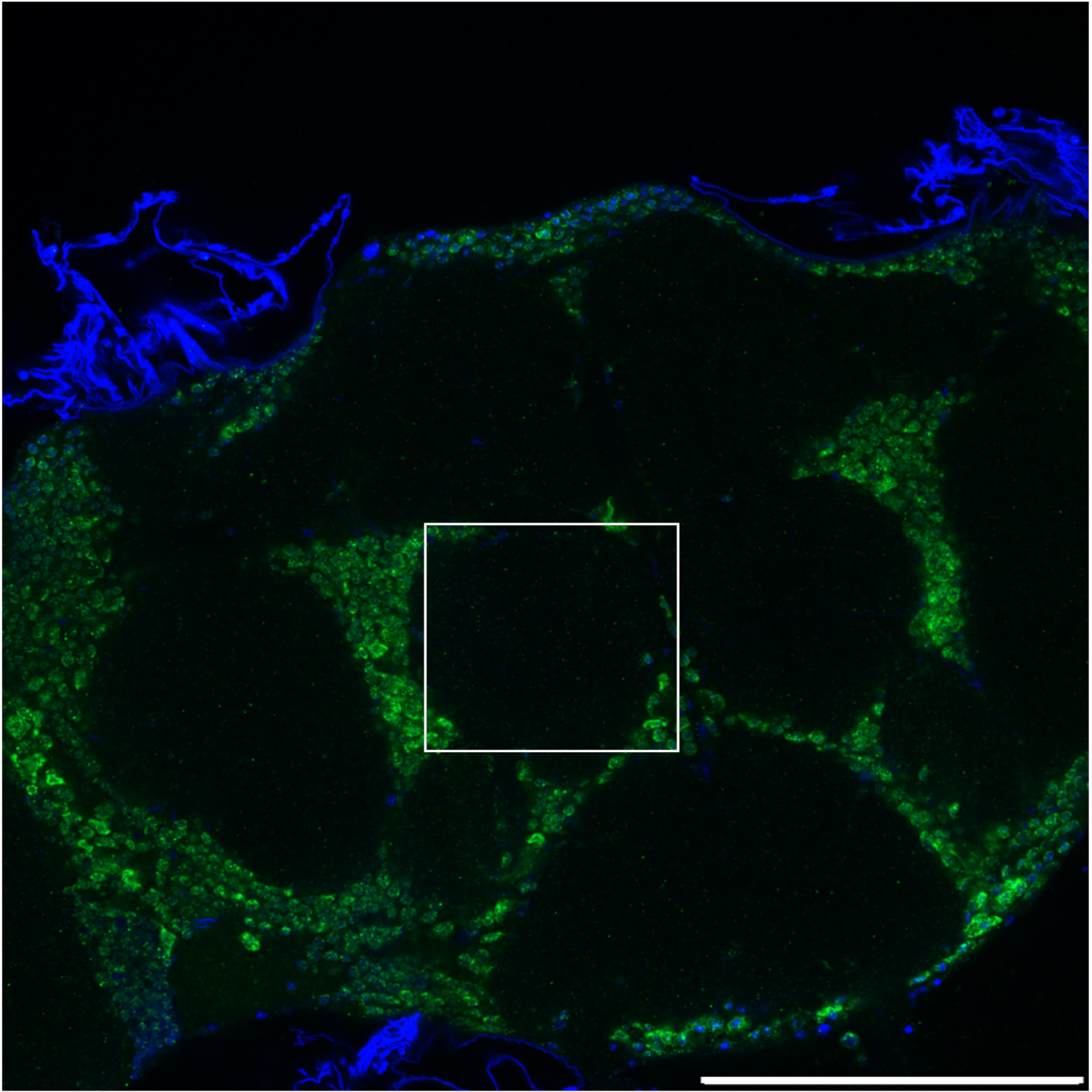

Supplement: Supplementary file 1 [file cells-12-00318-s001.zip › Figure S5.tif]

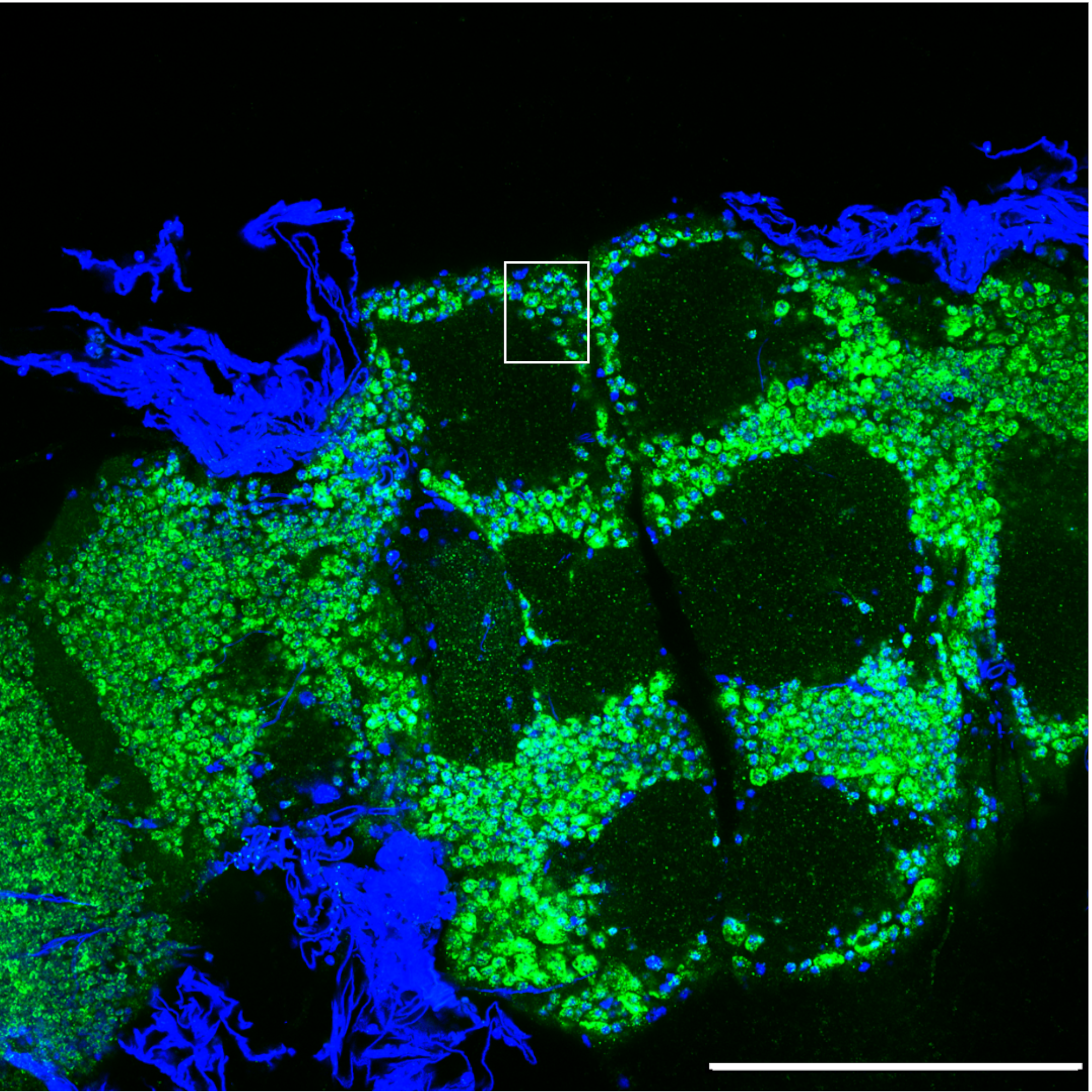

Supplement: Supplementary file 1 [file cells-12-00318-s001.zip › Figure S6.tif]
